# Optimised methods (SDS/PAGE and LC‐MS) reveal deamidation in all examined transglutaminase‐mediated reactions

**DOI:** 10.1002/2211-5463.12575

**Published:** 2019-01-18

**Authors:** Éva Sivadó, Meddy El Alaoui, Robert Kiraly, László Fesüs, Frédéric Delolme, Adeline Page, Saïd El Alaoui

**Affiliations:** ^1^ Research Department Covalab S.A.S Lyon France; ^2^ Department of Biochemistry and Molecular Biology Faculty of Medicine University of Debrecen Hungary; ^3^ Protein Science Facility SFR BioSciences CNRS UMS 3444 Inserm US 8 ENS UCBL Lyon France

**Keywords:** deamidation, fluorescence detection, SDS/PAGE, transamidation, transglutaminases

## Abstract

Transglutaminases (TGs) are a family of structurally and functionally related enzymes that catalyse calcium‐dependent post‐translational modifications of proteins through protein–protein crosslinking, amine incorporation, or deamidation. For many years deamidation mediated by TGs was considered to be a side reaction, but recently substrate‐specific deamidations have been reported. Here we describe an optimised SDS/PAGE assay for the easy and rapid monitoring of the TG reaction with small peptides. The relative proportion of deamidation to transamidation was evaluated by densitometric analysis and confirmed by nano‐liquid chromatography–nano‐electrospray ionisation MS. We further investigated the effect of reaction conditions on transamidation and deamidation of TG1, TG2 and blood coagulation factor XIII A‐subunit (FXIII‐A) enzymes using a panel of glutamine‐containing peptide substrates. The ratio of transamidation to deamidation was enhanced at high excess of the acyl‐acceptor substrate and increasing pH. In addition, it was influenced by peptide substrates as well. Whereas deamidation was favoured at low cadaverine concentrations and acidic pH, no significant effect of calcium was observed on the ratio of transamidation/deamidation. Under our experimental conditions, deamidation always occurred *in vitro* even at high excess of the acyl‐acceptor substrate, and the reaction outcome was shifted to deamidation at neutral pH. Our results provide clear evidence of the deamidation in the TG reaction, and may serve as an important approach for *in vivo* analysis of deamidation to better understand the role of TGs in biological events.

**Abbreviations**
ESI: electrospray ionisation
FAM: fluorescein amidite
FXIII‐A: blood coagulation factor XIII A‐subunit
HPLC: high‐performance liquid chromatography
LC‐MS: liquid chromatography–mass spectrometry
PTM: post‐translational modification
TG: transglutaminase

Transglutaminases (TGs; EC 2.3.2.13) are widely distributed enzymes with pleiotropic functions. Nine members have been described in mammals: keratinocyte (TG1), tissue (TG2), epidermal (TG3), prostate (TG4), type 5 (TG5), neuronal (TG6), type 7 (TG7), blood coagulation factor XIII A‐subunit (FXIII‐A), and the catalytically inactive erythrocyte band 4.2 protein 1.

The distribution and physiological roles of TGs have been investigated in various cell types and tissues. TG2 is the most studied member of the TGs family. This multifunctional protein has diverse cellular localisation and is implicated in several physiological (regulation of cell survival/death processes, cell adhesion, migration, signal transduction, proliferation) and pathological processes (coeliac disease, neurodegenerative disorders, fibrosis, inflammatory diseases, metabolic diseases and cancer) 2. TG1 mainly exists in the upper spinous and granular layers of the skin; it is involved in the terminal differentiation of keratinocytes by the formation of the crosslinked cell envelope 3. Thrombin‐activated factor FXIII‐A plays an essential role in the stabilisation of fibrin clots and in wound healing through the formation of isopeptide bonds 4.

TGs catalyse Ca^2+^‐dependent post‐translational modifications of proteins by generating a protein–protein crosslink (between a specific γ‐carboxamide group of a glutamine and an ε‐amino group of a lysine side chain), amine incorporation or deamidation. In the first step active site cysteine reacts with the γ‐glutaminyl group of the proteins or peptides leading to the formation of a thioester intermediate. In the second step the acyl group is transferred to an amine substrate resulting in the formation of an isopeptide bond or the water can act as an alternative nucleophile leading to site‐specific deamidation of the glutamine residue 5, 6. Many of these crosslinking reactions occur within 5–10 min, for example in case of FXIII‐A between glutamine (Gln398 or ‐399) and lysine (Lys406) residues of the fibrin γ‐chains 7, whereas crosslinking of fibrin α‐chains involving Gln221, ‐237, ‐328, or ‐366 and Lys208 or ‐606 takes place more slower 4, 8.

Deamidation of glutamine and asparagine residues is one of the most prevalent post‐translational modifications and converts an uncharged amino acid to a negatively charged residue introducing alternations in the protein's conformation. The protein function can be changed as it is determined by its global structure, and electrostatic protein–protein interaction can be modulated as well 9. Deamidation can occur in different ways (non‐enzymatically, by glutamines or phosphate‐activated glutaminases) regulating several biological processes 10, 11, 12. Moreover, cytotoxic necrotising factors in *Escherichia coli* and necrotoxin in *Bordetella*, which are considered to be functional relatives of TGs, can induce the formation of stress fibres by the deamidation of Rho proteins 13.

Deamidation by TGs was believed to be a side reaction, taking place only in the absence of primary amines or at low pH when availability of amines is limited 5, but recently it has been reported that selective deamidation in small heat shock protein 14 and βB2‐ and βB3‐crystallins 15 can occur in a substrate‐specific manner. Respectively, the substrate affinity and reaction conditions can influence the propensity for deamidation and transamidation 16. Many research works are focusing on the examination of TG crosslinking activity, which is connected to some diseases such as fibrosis and neurodegeneration. So far TG‐mediated deamidation activity has been related to coeliac disease 17, 18, and only a few reports have been published on its role in other physiopathological processes 15, 19, 20, 21. These studies support the potential role of TG‐dependent deamidation in the regulation of biological processes.

In this study we report an optimised SDS/PAGE assay for the rapid and easy detection of both transamidated and deamidated peptides. TG1, TG2, FXIII‐A enzymes and a panel of glutamine‐containing peptides were examined to determine how the ratio of deamidation to transamidation can be influenced by some reaction parameters, such as affinity for substrates, amine donor concentration, Ca^2+^ concentration and pH. The identity and the relative quantity of the reaction products were confirmed by liquid chromatography–mass spectrometry (LC‐MS) analysis. Our results are well correlated with the published data, cited above. Unexpectedly, under our reaction conditions, we found that a particular deamidation always occurs *in vitro*, even at high excess of the acyl‐acceptor substrates.

## Materials and methods

### Materials

All materials were obtained from Sigma‐Aldrich (Lyon, France) except where otherwise indicated. Recombinant human TG2 (rhu‐TG2, cat. no. T002), recombinant human keratinocyte TG (TG1, Art. No. T009), and recombinant human blood coagulation factor XIII‐A (FXIII‐A, cat. no. T027) were purchased from Zedira (Darmstadt, Germany). Peptides were obtained from Covalab (Villeurbanne, France). MilliQ water for mass spectrometry analysis was obtained from an ELGA system (ELGA Labwater, Millipore, Lyon, France).

### Synthetic peptide substrates

The peptides and their derivatives [fluorescein amidite (FAM)‐labelled peptides and deaminated controls] were synthesis by Covalab according to the established method for the production of synthetic peptides using solid‐phase peptide synthesis described by Merrifield *et al*. 22. Their purity was determined by analytical and preparative reversed‐phase HPLC and mass spectrometry. All peptides were dissolved in DMSO at a final concentration of 10 mm. Isoenzyme‐specific glutamine‐containing peptides have been reported by Sugimura *et al*. 23, 24 and used to develop specific TG activity assays 25, 26. K9 is a natural TG2 sequence based on β‐casein 27. Among the natural reactive glutamine site chains in fibrinogen α‐chains a short peptide sequence containing Q238 8 was chosen for our experiments. The corresponding deamidated control peptides were synthesised by Covalab (Table 1).

**Table 1:** Sequence of the glutamine‐containing amine acceptor and deamidated control peptides

Peptides	Sequence
T26	5‐FAM‐^1^HQSYVDPWMLDH^12^‐CONH_2_
T26 (Q2E)	5‐FAM‐^1^HESYVDPWMLDH^12^‐CONH_2_
K9	5‐FAM‐^1^LGPGQSLVIG^10^‐COOH
K9 (Q5E)	5‐FAM‐^1^LGPGESLVIG^10^‐COOH
K5	5‐FAM‐^1^YEQHKLPDSWPF^12^‐COOH
K5 (Q3E)	5‐FAM‐^1^YEEHKLPDSWPF^12^‐COOH
F11	5‐FAM‐^1^DQMMLPWPAVAL^12^‐COOH
F11 (Q2E)	5‐FAM‐^1^DEMMLPWPAVAL^12^‐COOH
Fibrinogen αC	5‐FAM‐^325^TGNQNPGSPRPG^336^‐COOH
Fibrinogen αC (Q328E)	5‐FAM‐^325^TGNENPGSPRPG^336^‐COOH

### Transglutaminase reaction

Five micromolar of specific glutamine donor substrates labelled with fluorescent FAM and cadaverine (5–1000 μm) as an acyl acceptor were incubated with their corresponding isoenzymes at 37 °C; 18 mU·mL^−1^ of TG1, TG2, or thrombin (1 U·mL^−1^)‐activated FXIII‐A was applied in the assay buffer (20 mm Tris pH 7.2 or 8.0 or MES pH 5.0 or 6.0, 150 mm NaCl, 10 mm DTT) in the presence or absence of 10 mm EDTA. pH was 7.2, unless the effect of pH (5–8) was examined. The enzymatic reaction was initiated by the addition of 5 mm CaCl_2_, except in the experiments in which the effect of Ca^2+^ was investigated (0.1–5 mm).

### SDS/PAGE assay

The assay was previously reported by Kenniston *et al*. 28 and modified as briefly described below. After the incubation period the enzymatic reaction was stopped by boiling the sample in 6× SDS‐loading buffer (9.3 w/w DTT, 12 wt% SDS, 47 v/v% glycerol, 0.06 wt% bromophenol blue in 0.5 m Tris/HCl, pH 6.8). The reaction products were run on SDS/PAGE (15% T, 2.6% C; T represents the total concentration of polyacrylamide monomer expressed in g per 100 mL and C is the percentage of bis‐polyacrylamide) and visualised by fluorescence detection (Luminescent Image Analyzer LAS‐1000 plus; Fujifilm, Dusseldorf, Germany).

### Liquid chromatography–mass spectrometry analysis

The samples were diluted 10 times in a solution of 0.1% formic acid before analysis. Mass spectrometry analysis was performed on a linear ion trap LTQ Velos (Thermo Scientific, San Jose, CA, USA) with nano‐electrospray ionisation (ESI) source coupled in‐line to a nanoRSLC system Ultimate 3000 (Thermo Scientific, Germering, Germany). One microlitre of sample was injected via the autosampler. Samples were first desalted and concentrated on a reverse phase precolumn (C18 PepMap100, 300 μm i.d. × 5 mm, 5 μm, 100 Å; Thermo Scientific) for 3 min at 20 μL·min^−1^ with H_2_O/acetonitrile 98/2–0.1% formic acid. Samples were then separated on a nanocolumn (Acclaim C18, 15 cm × 75 μm i.d, 2 μm; Thermo Scientific). The HPLC gradient was 5–55% solvent B (A = 5% acetonitrile, 0.1% formic acid; B = 80% acetonitrile, 0.1% formic acid) for 30 min followed by 5 min 99% B. The total duration was set to 50 min at a flow rate of 300 nL·min^−1^. The oven temperature was kept constant at 40 °C.

MS spectra were recorded in the mass range *m*/*z* 500–1100 in positive ionisation mode; the enhanced scan rate was used for the full MS spectrum.

### Data analysis

Relative intensity of fluorescent bands was analysed with imagej and plotted with prism 5 (GraphPad Software Inc., San Diego, CA, USA) software.

## Results and discussion

### Determination of transamidation and deamidation rates by transglutaminases

Post‐translational modification (PTM) is one of the powerful regulatory elements that confer a specific function to each protein. Dysregulation of PTMs has been the object of a number of studies and was shown to be associated with several diseases 29. Among the different type of PTMs, deamidation is unique as it has been shown to occur in different ways: spontaneously, chemically and enzymatically 30.

Deamidation is the conversion of selected glutamine and also asparagine residues into glutamate and aspartate/isoaspartate through hydrolytic reaction. Such reaction requires only water, is conditioned by both sequence and structure, and is facilitated by physico‐chemical conditions such as high temperatures, extreme pH or high ionic strength.

Deamidation of specific proteins such as adrenocorticotropin 31 and lens crystallins 32 has been shown to have biological repercussions due to folding changes and/or modification of life‐span. Although some proteins can undergo the deamidation reaction with little or no loss of biological activity, others do lose activity, which can be associated with a growing list of pathological phenomena such as age‐related, neurological and autoimmune diseases 33.

While *in vivo* deamidation of Asn to Asp was clearly established, TG‐mediated deamidation of Gln is not fully understood and rather it was believed to be only a side‐reaction, occurring when the second substrate is not available or at acidic pH. However, over about a decade many reports focused on the study of the mechanism of hydrolysis of the specific Gln residues in order to elucidate the role of TGs in the physiopathology of some diseases and coeliac disease in particular.

In order to evaluate the deamidation reaction of TG to convert Gln to Glu, several methods were used depending on whether the Gln substrates were natural (e.g. proteins) or synthetic (e.g. peptides). In the case of proteins, deamidations were analysed through molecular modelling and biological activity 34, 36 whereas synthetic peptides were analysed by various techniques such as mass spectrometry (MS and LC‐MS), size exclusion chromatography, capillary electrophoresis, HPLC, 2D gels and western blot 14.

**Figure 1:**
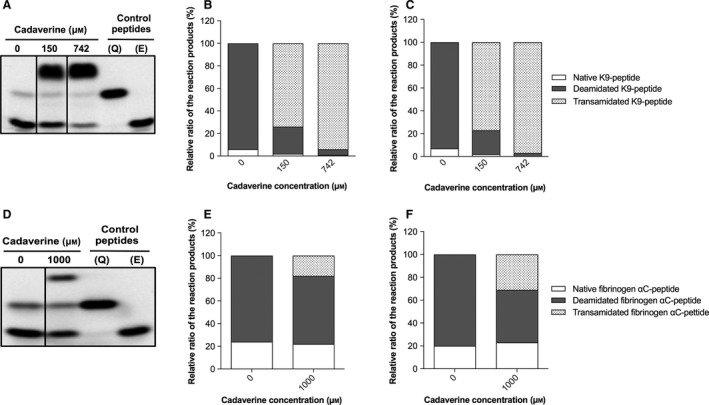
Relative quantification of TG reaction products using SDS/PAGE analysis and fluorescence detection. TG2 was incubated with FAM‐K9 peptide (A–C), thrombin activated FXIII‐A with FAM‐fibrinogen αC(325–336) peptide (D–F) and with cadaverine in the presence of Ca^2+^ for 20 min (TG2) or 5 h (FXIII‐A) at 37 °C. Q, reactive glutamine‐containing control peptide; E, deamidated control peptide. (B, E) Densitometry analysis of the SDS/PAGE. (C, F) Relative quantification of the reaction products by nano‐LC–nano‐ESI‐MS.

In this work we optimised an electrophoresis assay based on SDS/PAGE for the rapid and easy detection of both transamidated and deamidated peptides. All reaction products of FAM fluorescein‐labelled peptides could be simultaneously detected with high resolution and specificity. The relative ratio of deamidation and transamidation was evaluated by densitometric analysis (Fig. 1B,E). The identity and the proportion of the reaction products were confirmed by nano‐LC–nano‐ESI‐MS analysis. The relative quantities were calculated from extraction ions chromatogram areas of the doubly charged ions of each species (Fig. 1C,F, Figs S1, S2, Table S1), assuming that all the species have the same response factors. The data obtained by LC‐MS are in good correlation with the results of densitometric analysis, indicating that the optimised SDS/PAGE assay is a reliable method for the semi‐quantitative examination of both transamidated and deamidated reaction products.

### Catalytic activity of transglutaminases always generates deamidated peptide products

In this study TG1, TG2, FXIII‐A enzymes and five glutamine‐containing peptides (K5, T26, F11, K9 and αC(Q328)) (Table 1) were examined to determine how the ratio of deamidation to transamidation can be influenced by some reaction parameters such as substrates affinity, amine donor concentration, Ca^2+^ concentration and pH. K5 23, T26 and F11 24 were described as isoenzymes preferring glutamine substrates having high affinity to TG1, TG2 and FXIII‐A, respectively, whereas K9 27 and fibrinogen αC(Q328) peptides 8 are known as natural glutamine donor substrates for TG2 and FXIII‐A.

**Figure 2:**
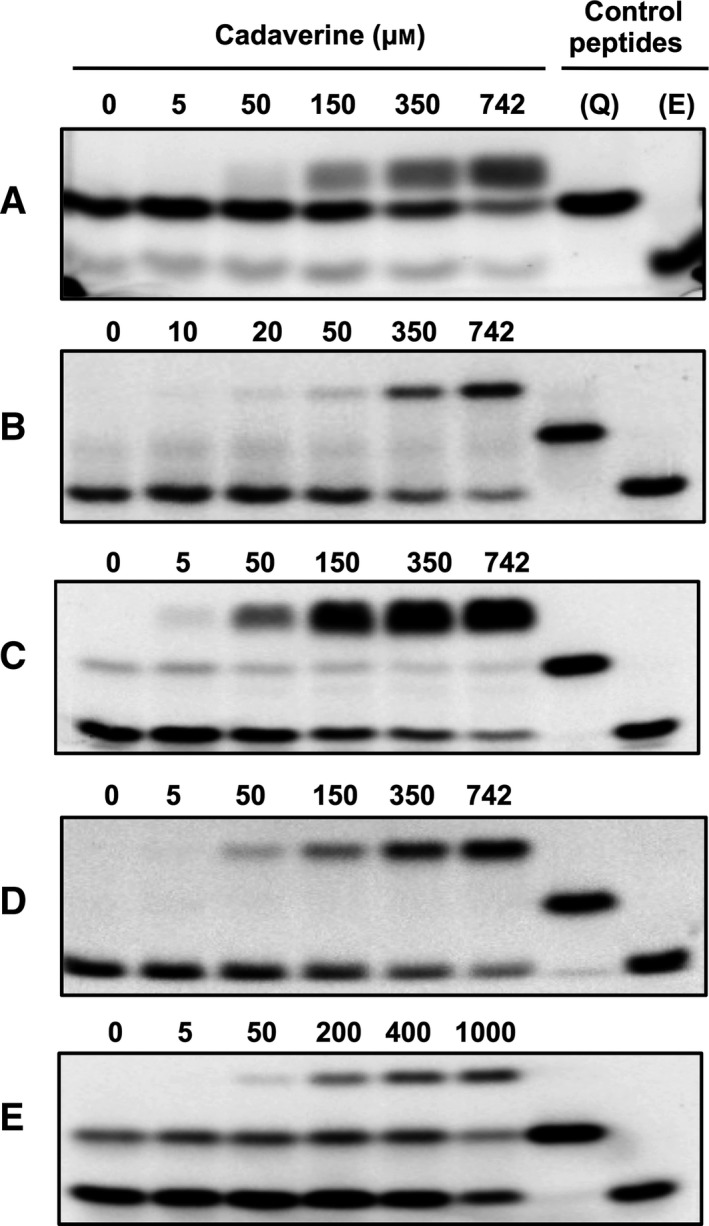
Monitoring of TG reaction at different cadaverine concentrations by SDS/PAGE analysis. TG1 was incubated with FAM‐K5 (A), TG2 with FAM‐T26 (B) and FAM‐K9 (C), thrombin activated FXIII‐A with FAM‐F11 (D), FAM‐fibrinogen αC(325–336) (E) glutamine donor peptides and with cadaverine in the presence of Ca^2+^ for 20 min (A–D) or 5 h (E) at 37 °C. The reaction products were separated by SDS/PAGE and visualised by fluorescence detection. Q, reactive glutamine‐containing control peptide; E, deamidated control peptide.

Applying high excess of the amine donor substrate (cadaverine), the ratio of transamidation to deamidation was increased, whereas deamidation was favoured at low cadaverine concentrations. In the absence of the second substrate, no crosslinked product was detected (Fig. 2). In addition this effect appeared to be substrate dependent and confirm the results obtained by the group of Sollid 16. TG2 transamidase activity was rather higher with K9 (Fig. 2B) than with T26 peptide (Fig. 2C) indicating better recognition of natural glutamine substrate. This difference was not observed with FXIII‐A as the reactivity is higher with F11 than with fibrinogen αC(Q328) peptides. Indeed upon the same reaction conditions the relative ratio of deamidated/transamidated fibrinogen αC(Q328) peptide was increased (Fig. 2E) whereas F11 peptide was preferred for transamidation (Fig. 2D). This slight difference in the deamidation/transamidation rate may be explained by the influence of the neighbouring amino acids relative to the targeted Gln and may affect the affinity of the enzymes. Indeed Boros *et al*. 15 reported that TG2 can process site‐specific deamidation, and a proline at position +2 to the specific glutamine residue may positively influence deamidation 35.

Moreover, the results reveal that TG2‐ and FXIII‐A‐catalysed substrate deamidation could always occur as an excess of primary amine did not completely inhibit deamidation (Fig. 1B–E). However, deamidation by TG1 is less obvious in all conditions studied and this could explain why K5 may not be good substrate of this enzyme for deamidation (Fig. 2A). Using natural and good substrate for TG1 will be of interest in completing this work.

### Effect of pH on the transglutaminase‐catalysed deamidation

**Figure 3:**
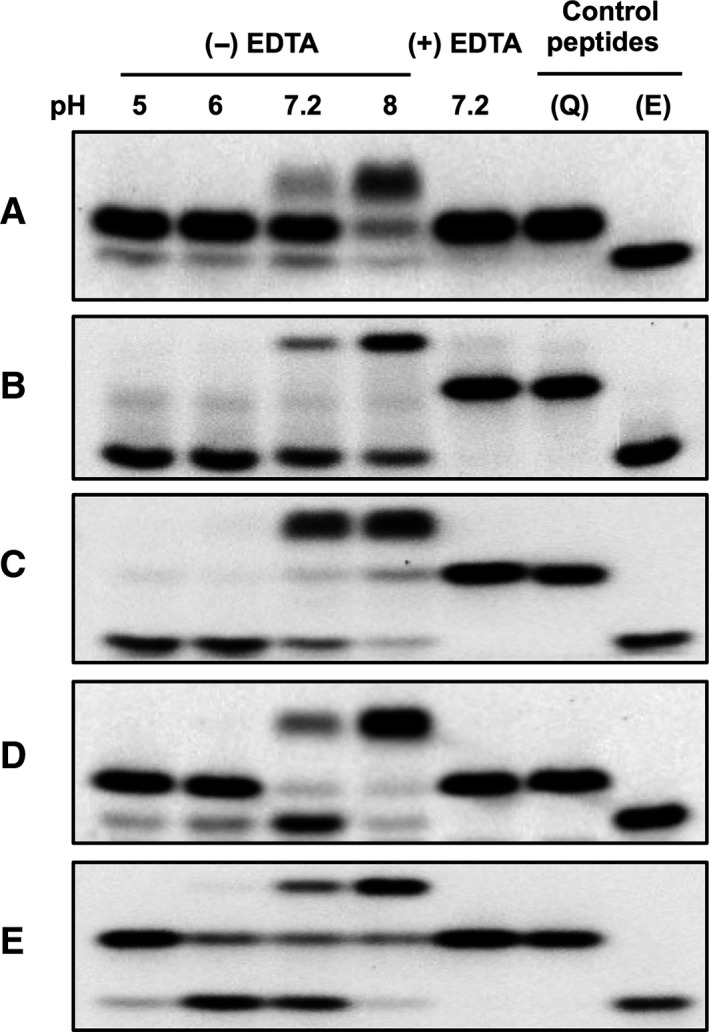
Monitoring of TG reaction at different pH values by SDS/PAGE analysis. TG1 was incubated with FAM‐K5 (A), TG2 with FAM‐T26 (B) and FAM‐K9 (C), thrombin‐activated FXIII‐A with FAM‐F11 (D) and FAM‐fibrinogen αC(325–336) (E) glutamine donor peptides and with cadaverine as acyl‐acceptor in the presence (−EDTA) or absence (+ EDTA) of Ca^2+^ at pH 5–8 for 20 min (A–D) or 5 h (E) at 37 °C. The reaction products were separated by SDS/PAGE and visualised by fluorescence detection. Q, reactive glutamine‐containing control peptide; E, deamidated control peptide.

Fleckenstein *et al*. 35 described that the TG2‐catalysed reaction of gliadin substrate is strongly influenced by pH. Here, we extended the investigation for two other isoenzymes and five peptide substrates as detailed in the Materials and methods (Fig. 3). In all cases the transamidation and deamidation reactions were analysed in buffers with pH values ranging from 5 to 8. TG1 (Fig. 3A) and FXIII‐A (Fig. 3D,E) seem to not be active at acidic pH, but at pH 6.0 fibrinogen αC(Q328) peptide was deamidated in the majority of cases (Fig. 3E). At pH 8.0 all the peptides were converted to transamidated forms by all the corresponding enzymes. In the condition of neutral pH, TG1 was not active due probably to the low kinetic reaction, whereas TG2 and FXIII‐A were able to convert the peptides to transamidated and deamidated products. The proportion of each product depends on the type of the enzyme and also the glutamine substrate. T26 was highly deamidated by TG2 whereas the natural substrate K9 was in the majority of cases transamidated (Fig. 3B,C) and with FXIII‐A the deamidation was more important than the transamidation for both substrates (Fig. 3D,E). These results further demonstrate that the rate of the transamidation reactions is significantly increased at alkaline pH indicating the importance of the nucleophily of the amine, which must be unprotonated. As the pKa of the cadaverine amine is around 10, it is expected that the transamidation reaction can be favoured over deamidation. Indeed in the report of Fleckenstein *et al*. 35 using 5‐(biotinamido)penthylamine as an amine donor with p*K*_a_ around 10.5, a general base‐catalysed deacylation mechanism was proposed for the transamidation reaction through a nucleophilic attack on the thiol ester intermediate. Increasing the pH to narrow the p*K*_a_ of the base would decrease its protonation, and consequently the competition by water molecules is blocked explaining the increase of transamidation rate.

### Effect of Ca^2+^ on the transglutaminase‐catalysed deamidation

**Figure 4:**
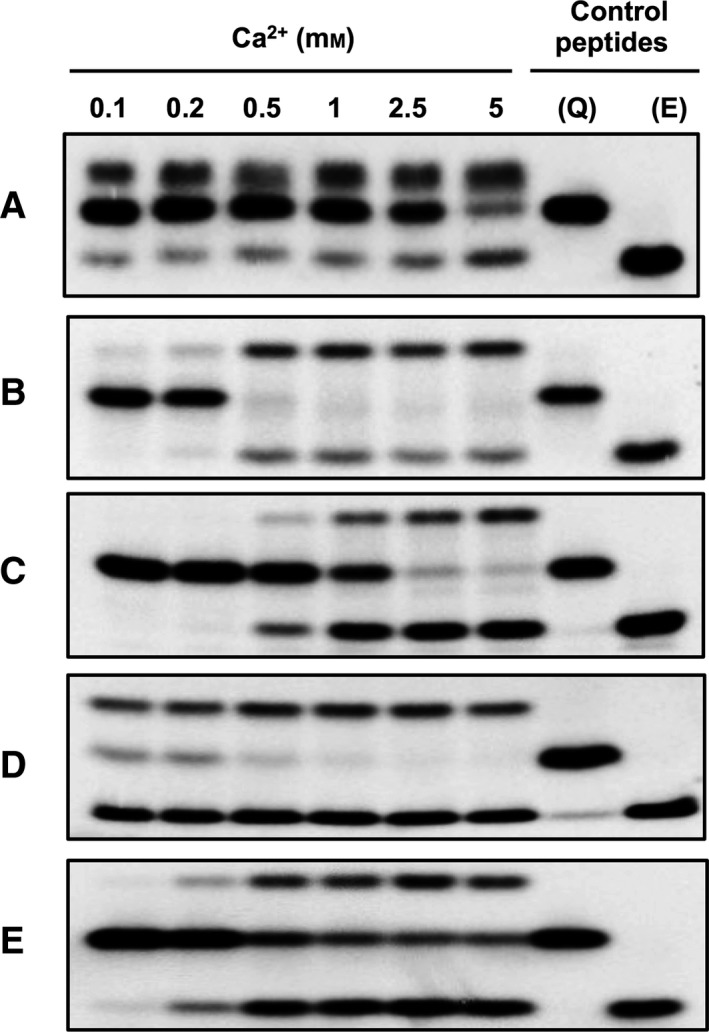
Monitoring of TG reaction at different Ca^2+^ concentrations by SDS/PAGE analysis. TG1 was incubated with FAM‐K5 (A), TG2 with FAM‐T26 (B) and FAM‐K9 (C), thrombin‐activated FXIII‐A with FAM‐F11 (D) and FAM‐fibrinogen αC(325–336) (E) glutamine donor peptides and with cadaverine as acyl‐acceptor at different Ca^2+^ concentrations for 20 min (A–D) or 5 h (E) at 37 °C. The reaction products were separated by SDS/PAGE and visualised by fluorescence detection. Q, reactive glutamine‐containing control peptide; E, deamidated control peptide.

It is well known that Ca^2+^ is required for the activation of TGs through inducing a large conformational change in the enzyme structure 37, 38. Because of the large difference in the open and closed conformations, we hypothesised that depending on Ca^2+^ binding, more transient conformers with a different hydrodynamic radius can be exhibited. Probably at lower Ca^*2+*^ concentrations the substrate‐binding channel could be particularly covered and the entrance of the second substrate could be inhibited resulting on the deamidation of the glutamine donor substrate via hydrolysis. Transamidation and deamidation were analysed with different Ca^2+^ concentrations ranging from 0.1 to 5 mm as described in Materials and methods (Fig. 4). At lower Ca^2+^ concentration (0.1–0.2 mm) TG2 seems to not be active (Fig. 4B,C) whereas TG1 converted the majority of K5 to transamidated product (Fig. 4A). With FXIII‐A we observed high activity with F11 substrate (Fig. 4D) and low activity with natural substrate (Fig. 4E), which can be explained by the high affinity of the enzyme to the synthetic substrate. By increasing Ca^2+^ concentration the enzymes were more active and both transamidation and deamidation occurred at a different rate depending on the Gln substrate: transamidation was observed more with TG1 and TG2 using K5 and T26 substrates, respectively, and more deamidation was obtained with TG2 and FXIII‐A using K9 and fibrinogen αC(Q328). Based on these results no significant effect of calcium on the ratio of transamidation/deamidation was obtained.

In conclusion, our assay based on SDS/PAGE provides an easy and rapid method for monitoring the TG reaction with synthetic small peptides. The fractions of native, deamidated and transamidated peptides can be successfully separated and their rates were confirmed by nano‐LC–nano‐ESI‐MS. Under our experimental conditions we noticed that a particular deamidation always occurs, even at high molar concentration (148–200‐fold molar excess) of amine donor substrate and it is preferred at low to neutral pH. These data provide further evidence for the simultaneous reactions of transamidation and deamidation, but further work will be needed to confirm this *in vitro* and *in vivo* at the endogenous proteins level. Knowledge of the *in vivo* regulation of transamidation *versus* deamidation is therefore crucial for the elucidation of the TG enigma. Most of the TG activity assays are based on detection of the transamidated products. Since the deamidation is no longer believed to be a side reaction, TG activity cannot be accurately evaluated only by detecting the crosslinked products.

## Conflict of interest

The authors declare no conflict of interest.

## Author contributions

ES performed the majority of the experiments, analysed and interpreted the data and contributed to the writing of paper; MEA contributed to the experiments and writing of the paper; AP and FD performed LC‐MS analysis; RK and LF contributed to the writing of the paper; SEA conceived the project and contributed to writing of the paper.

## Supporting information


**Fig. S1.** Nano‐LC–nano‐ESI‐MS analysis transglutaminase reaction products eXtracted Ions Chromatograms (XIC). TG2 was incubated with FAM‐K9 peptide in the absence (a) or with 150 μm of cadaverine (b) in the presence of Ca^2+^ for 20 min at 37 °C. From top to bottom, XICs of *m*/*z* 649.84 from FAM‐K9 peptide native form, *m*/*z* 692.40 from FAM‐K9 peptide transamidated form and *m*/*z* 650.32 from FAM‐K9 peptide deamidated form (MA = peak area).
**Fig. S2.** Nano‐LC–nano‐ESI‐MS analysis of transglutaminase reaction products eXtracted Ions Chromatograms (XIC). Thrombin‐activated FXIII‐A was incubated with FAM‐fibrinogen αC(325–336) peptide in the absence (a) or with 1000 μm of cadaverine (b) in the presence of Ca^2+^ for 5 h at 37 °C. From top to bottom, XICs of *m*/*z* 770.36 from FAM‐fibrinogen αC(325–336) peptide native form, *m*/*z* 770.88 from FAM‐fibrinogen αC(325–336) peptide deamidated form and *m*/*z* 812.88 from FAM‐fibrinogen αC(325–336) peptide transamidated form (MA = peak area).
**Table S1.** Molecular mass and *m*/*z* (mass‐to‐charge ratio) of K9 and fibrinogen αC(325–336) glutamine donor peptides.
