# Supplementary material for: Optimised methods (SDS/PAGE and LC‐MS) reveal deamidation in all examined transglutaminase‐mediated reactions
Source: FEBS Open Bio. 2019 Jan 18;9(2):396–404. doi: 10.1002/2211-5463.12575 (PMC6356169; doi:10.1002/2211-5463.12575)
Supplement: Supplementary file 1 — Fig. S1. Nano‐LC–nano‐ESI‐MS analysis transglutaminase reaction products eXtracted Ions Chromatograms (XIC). TG2 was incubated with FAM‐K9 peptide in the absence (a) or with 150 μm of cadaverine (b) in the presence of Ca2+ for 20 min at 37 °C. From top to bottom, XICs of m/z 649.84 from FAM‐K9 peptide native form, m/z 692.40 from FAM‐K9 peptide transamidated form and m/z 650.32 from FAM‐K9 peptide deamidated form (MA = peak area). Fig. S2. Nano‐LC–nano‐ESI‐MS analysis of transglutaminase reaction products eXtracted Ions Chromatograms (XIC). Thrombin‐activated FXIII‐A was incubated with FAM‐fibrinogen αC(325–336) peptide in the absence (a) or with 1000 μm of cadaverine (b) in the presence of Ca2+ for 5 h at 37 °C. From top to bottom, XICs of m/z 770.36 from FAM‐fibrinogen αC(325–336) peptide native form, m/z 770.88 from FAM‐fibrinogen αC(325–336) peptide deamidated form and m/z 812.88 from FAM‐fibrinogen αC(325–336) peptide transamidated form (MA = peak area). Table S1. Molecular mass and m/z (mass‐to‐charge ratio) of K9 and fibrinogen αC(325–336) glutamine donor peptides. [file FEB4-9-396-s001.pdf]

Supplementary figures

Table S1

Peptides	Molecular Mass (Da)	m/z (charge)
K9 (native)	1298.43	649,.4 (2+)
K9 (deamidated)	1299.41	650.32 (2+)
K9 (transamidated)	1383.61	692,.0 (2+)
Fibrinogen αC (native)	1539.56	770.36 (2+)
Fibrinogen αC (deamidated)	1540.54	770.88 (2+)
Fibrinogen αC (transamidated)	1624.74	812.88 (2+)

Fig. S1

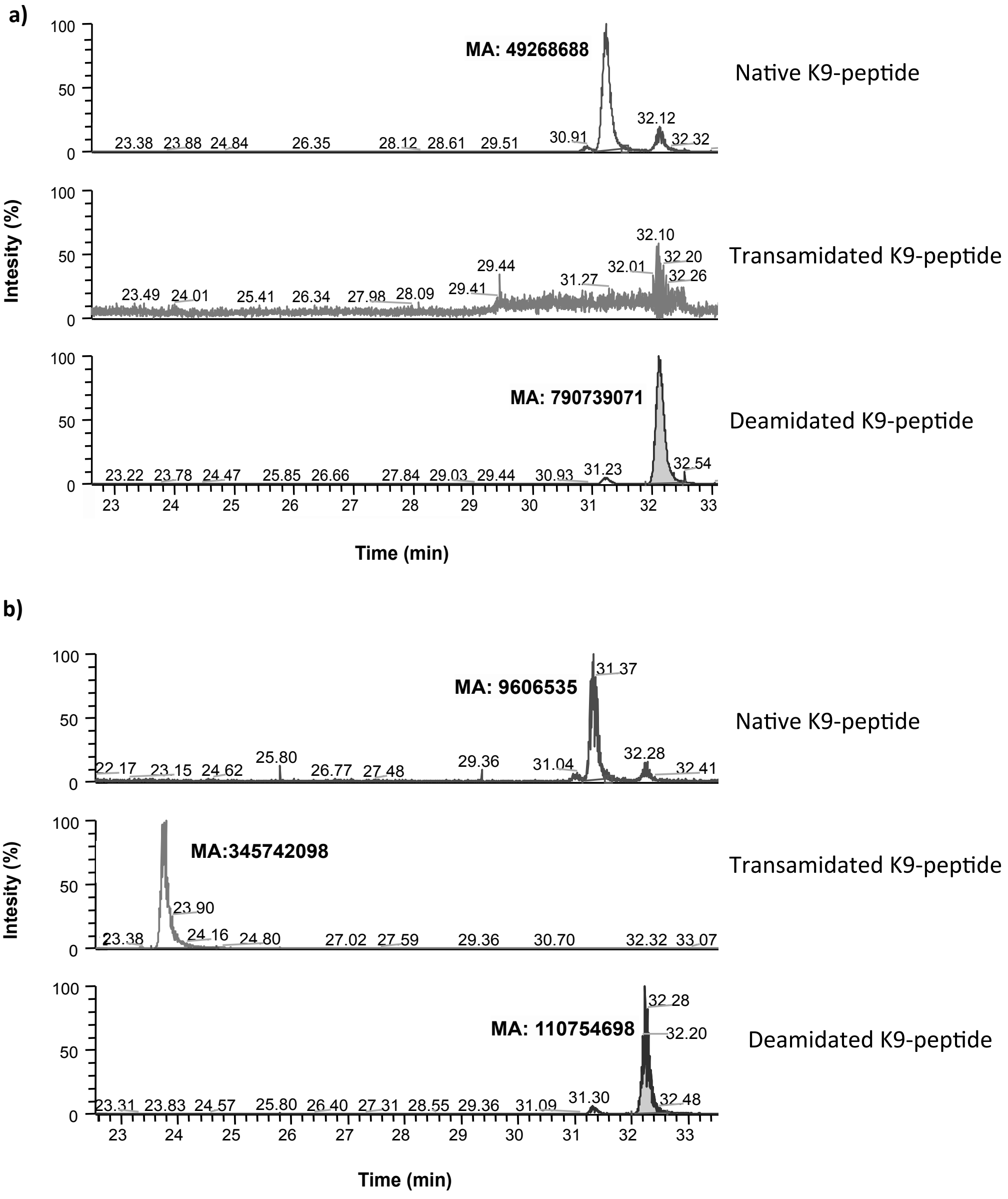

Fig. S2

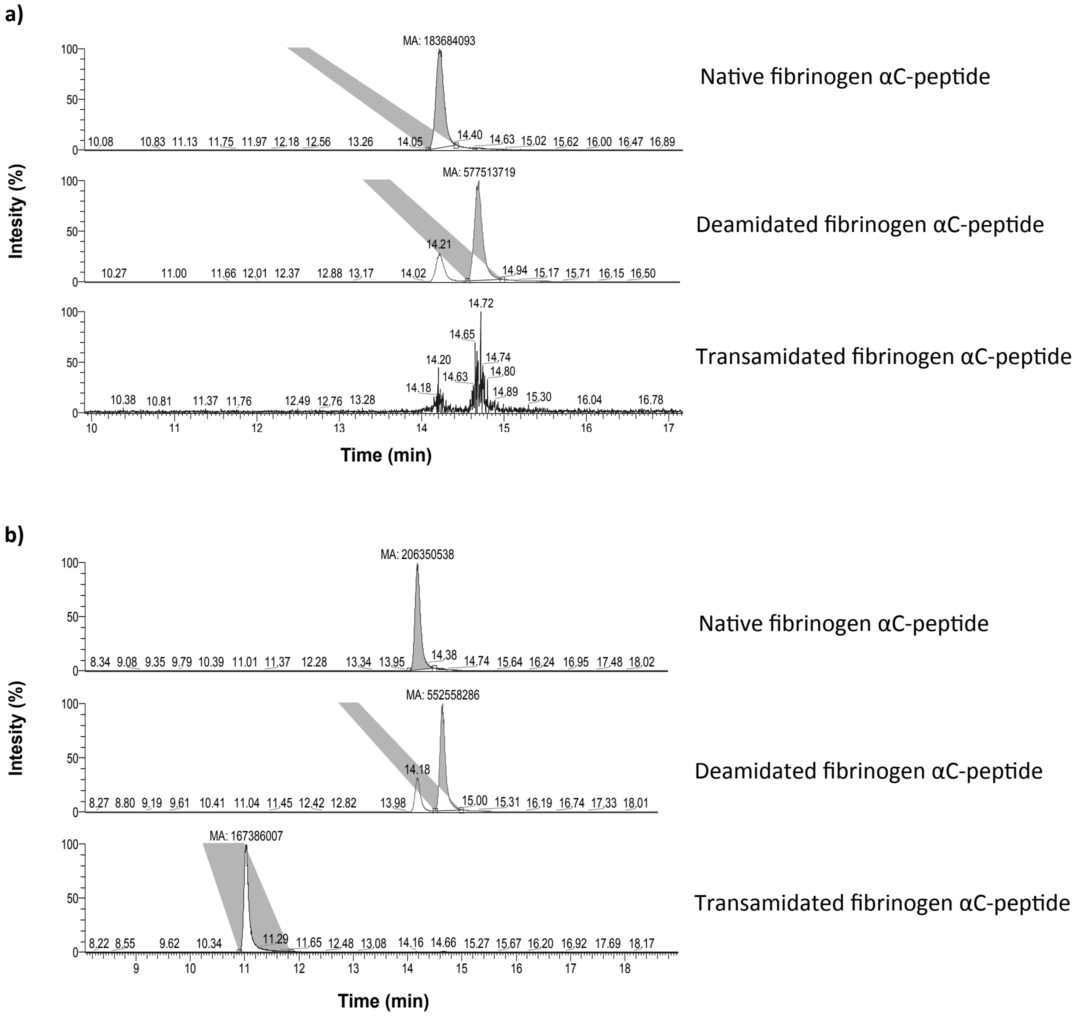
a)
MA: 183684093
Native fibrinogen αC-peptide
MA: 577513719
Deamidated fibrinogen αC-peptide
Transamidated fibrinogen αC-peptide
Intesity (%)
Time (min)
b)
MA: 206350538
Native fibrinogen αC-peptide
MA: 552558286
Deamidated fibrinogen αC-peptide
MA: 167386007
Transamidated fibrinogen αC-peptide
Intesity (%)
Time (min)
